# HIV-Associated Lymphomas: Updates from Pathogenesis to Treatment Strategies

**DOI:** 10.2174/011570162X367092250901062629

**Published:** 2025-09-04

**Authors:** Yi Liu, Jun Li, Yao Liu

**Affiliations:** 1 Department of Hematology-Oncology, Chongqing University Cancer Hospital, Chongqing, 400030, China

**Keywords:** HIV-associated lymphoma, pathogenesis, chemotherapy, targeted therapy, hematopoietic stem cell transplantation, HIV reservoirs

## Abstract

HIV-associated lymphoma (HAL) is an aggressive malignancy directly linked to HIV infection and accounts for more than 30% of cancer-related deaths in people living with HIV (PLWH). HAL subtypes, including diffuse large B-cell lymphoma (DLBCL), Burkitt lymphoma (BL), primary effusion lymphoma (PEL), and plasmablastic lymphoma (PBL), exhibit five to ten times higher incidence rates and distinct molecular profiles compared to HIV-negative lymphomas. Pathogenesis involves HIV-driven CD4^+^ T-cell depletion, chronic B-cell activation, and oncogenic viral coinfection. First-line therapy combines antiretroviral therapy (ART) with chemotherapy, achieving complete remission rates of 60-70% for DLBCL using R-EPOCH and 50-60% for BL with CODOX-M/IVAC. Relapsed/refractory cases show durable responses to CD19-CAR-T therapy; however, only 10% of HAL patients are enrolled in pivotal immunotherapy trials. Severe immunosuppression necessitates PET-CT-guided de-escalation and nanoparticle-based drug delivery systems to minimize toxicity. Emerging strategies include PD-1 inhibitors and broad-spectrum antivirals targeting HIV reservoirs, underscoring the need for precision medicine that integrates tumor genomics and viral dynamics.

## 1 INTRODUCTION

Acquired Immunodeficiency Syndrome (AIDS) is a chronic infectious disease caused by Human Immunodeficiency Virus (HIV) infection. With the development and widespread application of Antiretroviral Therapy (ART), the living conditions of people with AIDS have been significantly improved. However, it still poses a major challenge to global public health. HIV infects CD4^+^ T-lymphocytes, resulting in a significant reduction in CD4^+^ T-lymphocyte counts, which ultimately leads to a higher incidence of malignancies and opportunistic infections. With the widespread use of ART, AIDS-related opportunistic infections have been effectively controlled, and malignancies have become the leading cause of death among people living with HIV (PLWH) [1-3].

The latest WHO classification (5^th^ edition) refines the categorization of immunodeficiency-associated lymphoproliferative disorders (IDD-LPDs), encompassing subtypes such as post-transplant lymphoproliferative disorders (PTLD), HIV-associated lymphomas (HAL), primary immune deficiency-related LPDs, and iatrogenic immunosuppression-linked cases [4]. This integrated framework emphasizes clinicopathological heterogeneity, viral oncogenesis, and microenvi ronmental interactions, enabling the development of tailored therapies. The pathogenesis of HAL is relatively complex, in cluding the direct influence of HIV, immune system depletion, and immune escape, chronic activation of B cells, and co-infection with tumour-causing viruses. There have also been some advances in the treatment of HAL, including chemotherapy, monoclonal antibody therapy, and stem cell transplantation. The development of advanced antiviral materials includes heterocyclic compounds, metal-organic frameworks (MOFs), metal oxides, and carbon quantum dots (CQDs). These materials have become a cornerstone in addressing the challenges of HIV treatment, particularly in overcoming drug resistance and improving targeted delivery [5]. However, the treatment of HAL remains challenging, especially in relapsed or refractory cases. First-line treatment options also vary for different subtypes of HAL, and further research is needed to improve treatment, enhance patient survival, and improve quality of life.

This article systematically reviews the progress in diagnosis and treatment of HAL over the past five years using "HIV-associated lymphomas" as the key term in PubMed. It explores the pathogenesis of HAL and discusses the latest advances in its treatment, including various therapeutic strategies such as chemotherapy and immunotherapy, as well as the challenges faced in its clinical management. By gaining a deeper understanding of HAL’s pathogenesis and current clinical management strategies, we aim to explore more effective ways to manage this disease, to enhance patients’ prognosis and quality of life.

## 2 DEFINITION AND EPIDEMIOLOGY OF HIV- ASSOCIATED LYMPHOMA

### 2.1 Definition of HIV-associated Lymphoma

PLWH experience a higher burden of chronic comorbidities compared to the general population, even with ART. Chronic immune activation, inflammation, ART-related toxicities, and lifestyle factors drive this disparity. Among these comorbidities, cancer represents a critical area of concern.

PLWH face a significantly elevated risk of both AIDS-defining cancers (ADCs), such as Kaposi sarcoma (KS) and non-Hodgkin lymphoma (NHL), and non-AIDS-defining cancers (NADCs), including lung, anal, and liver cancers [6]. ADCs remain 10-50 times more common in PLWH than in HIV-negative individuals due to immunosuppression and oncogenic viral co-infections [6]. NADCs, such as anal squamous cell carcinoma (SCC), occur 1.5-3 times more frequently in PLWH, with higher rates linked to persistent HPV infection [7, 8]. In the U.S., 35% of anal SCCs in men and 2% in women occur in PLWH, reflecting HIV’s role in amplifying cancer risk [8].

With the effective control of HIV and the reconstruction of the immune system in PLWH, the incidence of HAL is gradually increasing, and it has become one of the most common malignancies among HIV-infected patients in developed countries [9]. Epidemiological data show that 95% of HIV-associated lymphomas are of B-cell origin, including diffuse large B-cell lymphoma (DLBL), Burkitt's lymphoma (BL), and primary effusion lymphoma (PEL) [10].

Other comorbidities, such as cardiovascular disease (CVD), are also more prevalent in PLWH. PLWH have a 1.5–2-fold higher risk of myocardial infarction and stroke, partly due to chronic inflammation [11, 12]. Non-infectious conditions like chronic kidney disease (3-4× higher risk) and nonalcoholic fatty liver disease (NAFLD) are exacerbated by HIV-related metabolic dysregulation [13, 14].

Accelerated aging and immune senescence further amplify comorbidity risks. PLWH develop age-related conditions 5-10 years earlier than the general population, with clonal hematopoiesis contributing to premature aging and cancer susceptibility [15, 16]. In summary, PLWH face disproportionate risks of cancer, CVD, and metabolic disorders, driven by immune dysfunction, viral co-infections, and chronic inflammation. Tailored screening and management strategies are essential to mitigate these disparities.

### 2.2 Epidemiology of HIV-associated Lymphomas

HIV-associated DLBCL is the most common subtype of HAL, accounting for approximately 45% of HAL [17]. The main pathophysiological features in these patients are persistent viremia and profound immunosuppression, with median CD4+ T-cell counts often below 200 cells/μL at diagnosis [18, 19]. BL is a rare and highly aggressive subtype of NHL, accounting for only 1 to 2% of all NHL cases. However, the incidence of BL is significantly increased in HIV-infected individuals, accounting for 25-40% of HAL. Notably, the incidence of HAL has not declined even with standardised use of ART [20]. Moreover, unlike other HALs, patients with HIV-associated BL often have CD4^+^ T-cell counts greater than 200 cells/μL [21], possibly due to the overactivation of B-cell germinal centers by CD4^+^ T-cells and the inhibition of B-cell apoptosis, which increases the likelihood of B-cell MYC translocations and the development of BL [22]. While Hodgkin lymphoma (HL) is not formally classified as an HIV-associated malignancy, its epidemiology in PLWH presents a striking paradox. Although HIV-related HL does not constitute a predominant clinical burden compared to other lymphomas, its incidence is elevated 5-15-fold in PLWH compared to the general population [23]. This epidemiological discrepancy has prompted significant research interest, particularly regarding its unique relationship with immune status. Notably, emerging evidence demonstrates a biphasic pattern of HL occurrence in PLWH, with incidence peaking at CD4+ counts of 150-199 cells/μL, followed by a decline with more severe immunosuppression [24, 25].

Primary central nervous system lymphoma (PCNSL) is a highly aggressive and rare tumour, but its incidence is significantly increased in HIV-infected patients, accounting for 12-15% of HAL [26]. 90-95% of PCNSL cases are DLBCL, with a small number of cases being BL or T-cell lymphomas [27]. About 75% of HIV-associated PBL cases exhibit EBV positivity [28]. Peripheral T-cell lymphoma (PTCL) and natural killer/T-cell lymphoma (NKTCL) are rare subtypes of NHL with only a small number of cases reported in HIV-infected individuals [29, 30]. Analysis of the Surveillance, Epidemiology, and End Results (SEER) database revealed that the incidence of HIV-associated cutaneous T-cell lymphomas had been increasing, from 0.09/100,000 in 2011 to 0.17/100,000 in 2017 [31]. In short, HALs are increasingly prevalent B-cell malignancies in HIV-infected individuals, with distinctive subtypes such as DLBCL and BL, and are closely linked to immune suppression and CD4^+^ T-cell counts.

## 3 PATHOGENESIS OF HIV-ASSOCIATED LYMPHOMA

### 3.1 Direct Carcinogenesis of HIV

HIV does not directly infect B cells; however, its viral proteins, including Nef, Tat, and p17, can enter bystander B cells, potentially leading to malignant transformation [32]. Notably, p17 alone can induce spontaneous B cell lymphomas in HIV transgenic mouse models [33]. Another study indicated that p17 variants rely on phosphatidylinositol-4,5-bisphosphate (PI-4,5-P2) to bind to protease-activated receptor 1 (PAR1), activating pathways that promote HAL [34, 35]. Tat enhances oncogene expression by activating the MYC promoter and causes chromosomal aberrations in B cells, contributing to lymphoma formation [36]. Furthermore, Tat can induce MYC-IGH oncogenic rearrangements, increasing HAL incidence [37]. Nef elevates activation-induced cytidine deaminase (AID) and MYC, further promoting genomic instability [38]. In contrast, chronic HIV infection fosters T-cell malignancy *via* the Vpr-VprBP-Plk4 complex and integration of HIV-1 provirus into key regulatory genes [39]. Overall, HIV drives B- and T-cell malignancies through diverse pathways, including protein interactions and genomic instability.

### 3.2 Inflammatory Response and Immune Activation

Chronic immune activation and sustained inflammatory responses are central to the pathogenesis of HAL. HIV infection disrupts the gut barrier, leading to microbial translocation [40], which releases bacterial products such as lipopolysaccharide (LPS) and perpetuates systemic inflammation [41]. This microbial translocation correlates with elevated soluble CD14 (sCD14) levels, a biomarker predictive of non-Hodgkin lymphoma risk (HR: 3.2) [42, 43]. Concurrently, HIV-driven overexpression of interleukin-6 (IL-6) activates the JAK/STAT3 pathway, promoting tumor angiogenesis and immune evasion [44]. Meanwhile, IL-17 enhances the production of pro-angiogenic factors, such as VEGF, in the tumor microenvironment, thereby accelerating lymphoma progression [45].

Persistent immune activation is characterized by upregulated exhaustion markers (PD-1, TIM-3) on CD8+ T cells (HR: 2.4) and aberrant activation of NF-κB and PI3K/AKT signaling pathways, directly driving B-cell proliferation and genomic instability [46]. Additionally, monocyte phenotypic remodeling in HIV-infected individuals exacerbates inflammatory cascades *via* the TNF-α/IL-1β axis [47], while gut dysbiosis further disrupts immune homeostasis [48]. Notably, the synergy between HIV and oncogenic *viruses* is amplified in the inflammatory milieu, where viral proteins LMP1 and vFLIP constitutively activate the NF-κB, thereby fostering lymphomagenesis [49, 50]. In summary, HAL pathogenesis arises from the intertwined effects of chronic inflammation, immune dysregulation, and microbiome disruption. Targeting inflammatory mediators or dual immune checkpoints may offer novel therapeutic strategies [50].

### 3.3 Immune Depletion and Immune Escape

HIV proteins and RNA can directly act on antigen-presenting cells such as macrophages and interfere with their functions, resulting in immune cells being unable to effectively recognize and present pathogen antigens, thereby reducing the effectiveness of the innate immune response [32]. HIV infection primarily targets CD4+ T cells and macrophages, leading to immunosuppression and abnormal cytokine secretion, which contribute to the development of HALs [51, 52]. HIV-infected macrophages play a critical role in maintaining HIV latency and facilitating disease progression through chronic inflammation. While bone marrow stromal antigen 2 (BST2), synthesized by macrophages, limits viral replication, the HIV viral protein U (Vpu) degrades BST2, allowing for persistent infection and inflammation [53, 54]. Additionally, macrophages harbor high levels of miRNA-99, which inhibits HIV degradation and promotes pyroptosis, thereby further amplifying chronic inflammation through the release of TNF-α, IL-6, and IL-1β [55, 56].

Indoleamine 2,3-dioxygenase (IDO), produced by antigen-presenting cells such as macrophages and dendritic cells, is upregulated by HIV proteins Tat and Nef, leading to Th17 cell depletion and the expansion of regulatory T cells (Tregs) [57-59]. This immune evasion is compounded by the upregulation of miRNA-146a, which dampens innate immune responses and downregulates CCL5 [56]. HIV also suppresses the activation of CD56bright NK cells, reducing their cytotoxicity against precancerous tissues [60]. CD4^+^ T cells exhibit a reduced quantity and function, while CD8+ T cells show increased expression of inhibitory receptors, such as PD-1, which impairs their effectiveness [61]. HIV also affects B cells by inhibiting NF-κB activity, resulting in reduced MHC class II gene transcription and an increased risk of marginal zone B lymphoma due to elevated B cell-activating factor (BAFF) [62, 63].

### 3.4 Co-infection with Oncogenic Viruses

EBV and HHV-8 are two common oncogenic viruses that are strongly associated with the development of HAL. Viral proteins encoded by EBV, such as latent membrane protein 1 (LMP1) and EBV nuclear antigen 1 and 2 (EBNA1, EBNA2), can induce the overexpression of the MYC oncogene, leading to the aberrant regulation of cellular signaling pathways, cellular metabolism, and cellular growth [20]. HHV-8 infection is also closely associated with the development of HAL. HHV-8 produces proteins such as viral interferon regulatory factor (vIRF), latency-associated nuclear antigen (LANA), and viral cell cycle proteins, as well as altering the DNA methylation profile of B cells, which promotes the malignant transformation of B cells [20, 64, 65]. Due to the interaction between HIV and HHV-8, virological indicators can provide some insight into the outcome and prognosis of patients with HAL. Studies have focused on assessing the prognostic value of various viral markers, including HIV viral load, EBV serostatus, and HHV-8 serostatus in HAL patients receiving CHOP chemotherapy [66]. High HIV viral load and positive HHV-8 serostatus were associated with poorer overall survival, whereas EBV serostatus showed no significant impact on HAL prognosis [66].

**Fig. (1):**
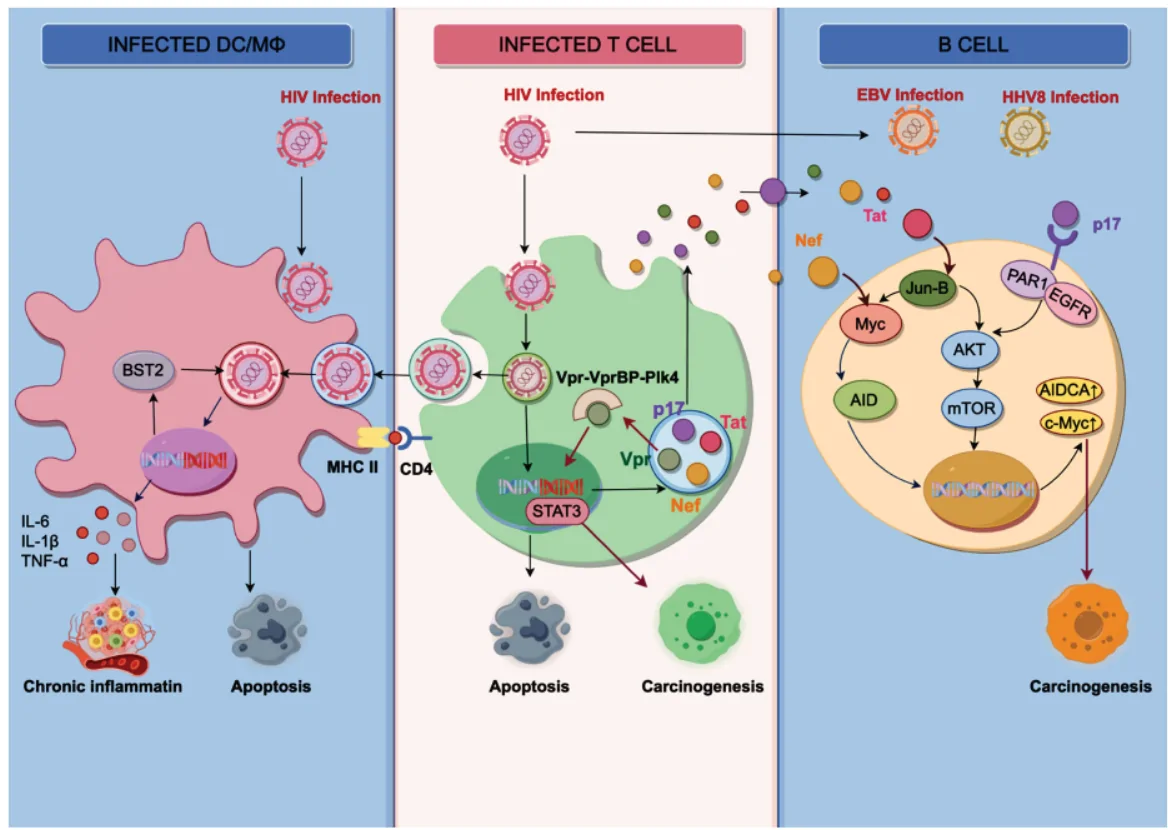
Pathogenesis of HIV-associated lymphoma. After HIV infects T cells, it produces viral proteins, such as Vpr, Tat, P17, and Nef. Vprinduces malignant transformation of T cells by forming Vpr-Vpr complexes that activate STAT3. Additionally, HIV can infect macrophages, promoting the secretion of IL-6, IL-1β, and TNF-α, which leads to chronic inflammation and creates an immunosuppressive microenvironment that supports lymphoma development. The Nef and Tat proteins secreted by HIV-infected cells can enter B cells, activating MYC and Jun-B, respectively, to promote oncogene expression in B cells. Meanwhile, p17 binds to the PAR1 receptor on the surface of B cells, activating the EGFR/AKT/mTOR pathway to facilitate malignant transformation of B cells.

HAL arises from complex mechanisms that involve both direct carcinogenesis and immune evasion. HIV proteins, such as Nef, Tat, and p17, interact with B cells, inducing malignant transformation and promoting genomic instability. Furthermore, HIV infection leads to immune depletion, particularly targeting CD4+ T cells and macrophages, which impairs antigen presentation and immune responses, and promotes co-infection with oncogenic viruses, contributing to HAL pathogenesis (Fig. **1**).

HIV-associated lymphoma (HAL) shares core oncogenic mechanisms with sporadic lymphomas, including reliance on viral oncoproteins and genomic instability resulting from mutations in MYC and TP53 [49]. However, it diverges through HIV-specific drivers that synergistically accelerate lymphomagenesis. Unlike sporadic cases, HAL exhibits direct viral carcinogenesis *via* HIV Tat/Nef proteins, inducing MYC-IGH rearrangements and p17-mediated DNA repair impairment [50]. It is further distinguished by unique inflammatory triggers, including gut dysbiosis-driven IL-6/IL-17 overexpression and macrophage pyroptosis, which sustain TNF-α/IL-1β storms [44, 47]. Immune dysregulation in HAL also differs markedly, characterized by an IDO-mediated Th17/Treg imbalance and CD56bright NK cell depletion, rather than the conventional PD-1/CTLA-4 upregulation [46]. These intersecting mechanisms include HIV-driven genomic hits, chronic inflammation, and immune exhaustion-position as a paradigm of accelerated malignancy, where persistent viral replication and immune activation remodel the microenvironment to amplify oncogenic stress.

## 4 PROGNOSIS AND ASSESSMENT OF HIV-ASSOCIATED LYMPHOMA

HAL is a highly heterogeneous group, and its typing and prognostic assessment can provide a basis for the development of individualised treatment, which is important for optimising patients' treatment options and improving survival quality and prognosis. Although the International Prognostic Index (IPI) score is a widely used prognostic tool for NHL patients, due to the highly heterogeneous genetic characteristics of HAL and clinical features that distinguish HAL from other NHLs, the IPI score has limitations in prognosticating in the HAL population. There is an urgent need to find a new type of prognostic indicator that is accurate, reliable, and accessible. Table **1** summarises the prognostic indicators of HAL and their clinical value, which, when combined with the IPI score, have improved the accuracy of the IPI prognostic model to varying degrees. In HAL, in addition to molecular markers, other factors may also affect patient prognosis, such as immune status and microenvironmental factors. The combined consideration of these factors will help establish a more accurate prognostic model. Future studies should focus on integrating these factors into comprehensive prognostic models to enhance risk stratification and guide personalized therapy for HAL patients.

## 5 THERAPY FOR HIV-ASSOCIATED LYMPHOMA

Treatment options for HAL include chemotherapy, monoclonal antibodies, and stem cell transplantation. First-line treatment options vary according to different subtypes of HAL, including R-EPOCH, R-CHOP, CODOX-M/IVAC, *etc*. [74]. For relapsed or refractory HAL, second-line salvage chemotherapy or transplantation may be considered. Table **2** lists the treatment options for different subtypes of HIV-associated lymphoma by therapeutic category. Although the treatment of HAL remains challenging, new therapeutic approaches offer hope for future research and treatment.

### 5.1 Diffuse large B-cell lymphoma

HIV-associated DLBCL is the most common HIV lymphoma and is highly heterogeneous. The cell-of-origin (COO) classification, the most common molecular subtyping method, categorizes lymphomas based on transcriptional profiles that mimic normal B-cell development. It divides DLBCL into germinal center B-cell (GCB) and non-GCB subtypes [75]. This classification is vital for prognosis and treatment planning, as GCB and non-GCB DLBCL subtypes exhibit distinct biological behaviors and drug responses, underscoring their value in personalized medicine [76]. Patients' responses to treatment and prognosis vary according to COO classification, EBV infection status, and CD4+ T-cell counts. ABC-type HIV-associated DLBCL is usually EBV-positive, whereas GCB-type HIV-associated DLBCL is usually EBV-negative [77, 78]. EBV-negative HIV-associated DLBCL cases exhibit significantly higher copy number alterations compared with EBV-positive cases [79]. In contrast, EBV-positive HIV-associated DLBCL cases exhibit lower CD4+ T-cell counts, lower mutation loads, and more frequent STAT3 mutations [77].

Unlike non-HIV-associated DLBCL, for which RCHOP is recommended as first-line treatment, the preferred regimen for the first-line treatment of HIV-associated DLBCL is the R-EPOCH regimen, as recommended in the 5th edition of the National Comprehensive Cancer Network (NCCN) guidelines for 2023, with the other recommended regimens being the RCHOP regimen [74]. Numerous studies have shown that HIV-associated DLBCL does not respond significantly differently to standard chemotherapy compared to diffuse large B-cell lymphoma in immunocompetent populations. In HIV-associated DLBCL, dose-adjusted (DA)-EPOCH has a 3-year OS rate of 71% and a PFS of 79% [80]. In contrast, the standard-dose EPOCH yields a 3-year OS rate of 77% and a PSF of 69%. There is no significant difference in efficacy between the two, both of which can be used as alternatives to CHOP in higher-risk groups [80]. In addition, intrathecal (IT) methotrexate for central nervous system (CNS) prophylaxis is increasingly emphasized in HIV-associated DLBCL as HAL is more prone to CNS invasion [81].

As the vast majority of HIV-associated DLBCL expresses CD20, the use of rituximab in combination with CHOP or EPOCH regimens has been shown to improve patient survival, with 5-year progression-free survival (PFS) rates as high as 87.8% and a 50% reduction in mortality risk. However, rituximab use requires monitoring of CD4^+^ T-cell counts, as studies have shown that its administration in individuals with CD4+ T-cell counts below 50 cells/μL significantly increases the risk of infection and death [82]. In addition, rituximab may trigger HBV reactivation, necessitating prophylactic antiviral therapy in high-risk groups and long-term HBV DNA monitoring in low-risk populations [83]. As high-intensity chemotherapy is more likely to cause myelosuppression, short-course (4-6 cycles) R+EPOCH regimens are a safer and more effective option than standard-dose R+DA-EPOCH, achieving comparable efficacy while reducing treatment-related deaths and adverse events (AEs), thus better balancing outcomes and tolerability [84, 85]. Other short-course chemotherapy regimens, such as three cycles of R2 (double-dose rituximab)-EPOCH, can also benefit patients with HIV-associated DLBCL, with 5-year PFS and overall survival (OS) rates of 64.3% and 71.8%, respectively [84].

In the era of new drugs, an increasing number of medicines are being tried for the treatment of HIV-associated DLBCL. Vorinostat (VOR), a histone deacetylase inhibitor, enhances the efficacy of the CD20 monoclonal antibody and chemotherapeutics, induces the lysis of EBV+ or HHV-8+ tumor cells, and reactivates latent HIV, which is expected to clear the HIV reservoir [86, 87]. Although previous studies have shown VOR combined with R-EPOCH to be safe and effective in primary HIV-associated DLBCL [88], it has not demonstrated a clear efficacy advantage in larger studies and is accompanied by more adverse events. No significant differences in 3-year objective response (OR) and PFS rates were found between the VOR+R-EPOCH *versus* R-EPOCH regimens (70% *vs*. 77%; 63% *vs*. 69%), and more grade 4 neutropenia and grade 4 thrombocytopenia were observed in VOR+R-EPOCH (47% *vs*. 20%; 29% *vs*. 2%) [80].

Although first-line treatment regimens are effective for most HIV-associated DLBCL patients, about 40% of patients still experience relapse or disease progression after receiving first-line therapy. The PD-1 inhibitor pembrolizumab, used as a second-line treatment, not only targets PD-1 high-expressing HIV-associated tumors but also promotes immune system recovery, demonstrating potential efficacy in this population [89]. According to National Cancer Institute, among four HIV-associated DLBCL patients treated with pembrolizumab monotherapy, two out of four patients achieved partial remission (PR) after 6 cycles of treatment, one had stable disease (SD) after 7 cycles, another ones died from bone marrow infiltration after three cycles of treatment and died from bone marrow infiltration. This study provided preliminary evidence of the safety of pembrolizumab in patients with relapsed, refractory HIV-associated DLBCL and demonstrated that partial remission was observed even in patients with CD4^+^ T-cell counts <50 cells/μL [90].

### 5.2 Burkitt Lymphoma

BL is a rare and highly aggressive B-cell NHL that accounts for less than 5% of lymphoma cases, with a higher incidence in children than in adults [91]. BL is chemotherapy-sensitive, grows rapidly, and has a propensity for CNS. Therefore, intensive chemotherapy regimens are often used in conjunction with supportive care to mitigate AEs. The latest edition of the NCCN guidelines for first-line treatment of HIV-associated BL recommends CODOX-M/IVAC or DA-EPOCH-R regimens, followed by R-Hyper CVAD [74].

No significant difference was observed between CODOX-M/IVAC and DA-EPOCH-R in HIV-associated BL. The 2-year PFS rate and OS rate of CODOX-M/IVAC regimen in HIV-phase BL were 55% and 55%, respectively. Studies have shown that the addition of rituximab to the CODOX-M/IVAC regimen increased the 2-year PFS rate to 81% and OS rate to 72% with no increase in treatment-related toxicities [92]. In addition, the dose-reduced CODOX-M/IVAC-R regimen in combination with intrathecal chemotherapy reduced the incidence of grade 3/4 toxic reactions to 79%, with a 1-year PFS rate of 69%, an OR rate of 72%, and a 2-year OS rate of 69% [93]. Taken together, R-CODOX-M/IVAC and the dose-reduced CODOX-M/IVAC-R regimen with intrathecal chemotherapy are effective strategies for HIV-associated BL, improving survival with acceptable toxicity.

DA-EPOCH-R and R-HyperCVAD are also recommended first-line treatment options. A retrospective analysis of HIV- and non-HIV-infected BL found that the response of HIV-associated BL to R-Hyper CVAD therapy was not significantly different from that of regular BL, with a 5-year OS rate of 27% for HIV-associated BL compared to 28.5% for regular BL [94]. Although R-Hyper CVAD has fair clinical efficacy, it is often limited by its drug toxicity [92]. Studies have shown that R-Hyper CVAD has an early chemotherapy-related toxicity event rate of 61.5% and the highest rates of serious infections (5.3%), bacteremia (9.2%), and CMV infection (7.9%) [95].

Given the poor tolerance of high-intensity chemotherapy in the pediatric and elderly populations, intermittent low-dose CHOP regimens or R-CyBorD regimens may be considered in HIV-associated BL. Previous studies have shown an OR rate of 47.8% in relapsed Burkitt lymphoma treated with intermittent low-dose CHOP, which was significantly higher than that of the intensive chemotherapy group (OR rate 19.0%) [96]. A case report showed that in children with HIV-associated BL, complete remission was achieved after 12 cycles of intermittent low-dose CHOP chemotherapy, and all biochemical parameters of the children were normal during the medication with no treatment-related adverse effects [97]. In contrast, in HIV-associated BL in adults, an intermittent low-dose chemotherapy regimen with R-CyBorD resulted in an OR rate of 78%, a treatment-related mortality rate of 6%, and a 6% incidence of treatment-related adverse events [91]. Thus, intermittent low-dose chemotherapy shows therapeutic potential as a relatively mild yet effective alternative treatment strategy in HIV-associated BL.

CNS involvement occurs in 30%-36% of HIV-associated BL, significantly higher than the 15.4%-16% in immunocompetent patients [98, 99], necessitating CNS prophylaxis [100]. In addition to susceptibility to CNS involvement, HIV-associated BL is more prone to relapse compared to BL in immunocompetent populations, with a relapse rate of approximately 10%-15% [84]. Second-line treatment options available for HIV-associated BL relapse include R-ICE, DA-EPOCH-R, R-GDP, and R-IVAC, which are often ineffective. In addition to chemotherapy alone as a second-line treatment for relapsed/refractory HIV-associated BL, the efficacy of AHSCT as a consolidation of first-line treatment and as a second-line treatment has been demonstrated to be precise, with patients achieving 3-year PFS and OS rates of 63% and 66%, respectively [84, 101].

### 5.3 Primary Effusion Lymphoma

PEL is a rare and aggressive B-cell tumour that accounts for approximately 4% of all HAL [102]. HIV-associated PEL has a poor prognosis compared to other HAL, with a median survival of only 10-22 months [103]. The preferred regimen for first-line treatment, as recommended by the NCCN guidelines, is the R-EPOCH regimen, with alternative recommendations for the R-CHOP regimen [74]. It is noteworthy that although HIV-associated PEL is often CD20-negative, the NCCN guidelines still recommend the use of rituximab. This is partly because most patients with PEL also have multicentric Castleman disease (MCD), for which rituximab is the standard of care [104, 105]. On the other hand, rituximab targets the KSHV-infected B-cell reservoir and reduces the release of inflammatory factors that drive the natural course of PEL [105]. Therefore, rituximab is recommended as a first-line treatment option for HIV-associated PEL. However, clinicians need to be vigilant for the development of Kaposi sarcoma during rituximab treatment [106]. Previous studies have shown that the CR rate of HIV-associated PEL patients to CHOP regimens was only 10%. In contrast, the CR to intensive regimens such as MTX, ACVBP, and ACVBP was 14.3%, suggesting that strong chemotherapy regimens may be more effective for PEL patients compared to CHOP regimens. However, for HIV-associated PEL patients with poor immune reconstitution, even with intensive regimens, it is difficult to achieve remission [103, 107, 108].

Immunomodulatory drugs (IMiDs), such as pomalidomide and lenalidomide, can downregulate the overexpression of IRF4 in PEL and reverse KSHV-induced downregulation of MHC-1 and ICAM-1 [109, 110]. A phase I study (NCT02911142) explored the safety and efficacy of the EPOCH-R2 regimen in HIV-associated PEL [111]. The study included 6 patients with HIV-related PEL who were treated with 6 cycles of EPOCH-R2 with thromboprophylaxis, opportunistic infection prophylaxis, and ART. The PR rate of HIV-associated PEL was 83.3% after 2 cycles, the CR rate was 50% after 6 cycles, the 2-year OS was 66.7%, and there was no significant decrease in CD4+ T-cell counts before and after the treatment [111]. The most common AE during EPOCH-R2 treatment was haematological toxicity (100%), followed by pulmonary embolism (50%) [111]. These preliminary results suggest that EPOCH-R2 may be an effective and relatively safe option.

Brentuximab vedotin (BV), an antibody–drug conjugate (ADC) targeting CD30, combines an anti-CD30 monoclonal antibody with a cytotoxic agent. Case reports indicate that BV has dual effects in HIV-associated PEL: controlling lymphoma progression and suppressing HIV infection [112]. One report described a patient with HIV-associated PEL who had a CD4 count of 29 cells/µL and an HIV viral load of 327,367 copies/mL prior to treatment [112]. After 6 courses of BV combined with antiretroviral therapy, the patient achieved complete remission (CR). Following 17 courses, the CD4 count increased to 448 cells/µL, and the HIV viral load dropped below the limit of detection. Although grade 2 sensory peripheral neuropathy developed during treatment, dose reduction of BV successfully reversed this adverse effect [112]. The mechanism of BV-targeted HIV clearance may be based on the frequent overexpression of CD30 by HIV latently infected CD4+ T cells in the suppressive AR environment. In vitro experiments have demonstrated that brentuximab vedotin significantly reduces the number of cells from infected individuals and the total amount of HIV-1 DNA in peripheral blood mononuclear cells in an ART-suppressive environment. This result supports the possibility that BV may have a positive effect on the clearance of latent HIV infection [113].

### 5.4 Plasmablastic Lymphoma

PBL is a rare subtype of NHL that is associated with HIV infection in 63% of cases [114] and often expresses plasma cell markers such as CD138, CD38, and MUM1/IRF4 [115-117]. Although HIV infection status causes PBL to present at a later stage, it does not affect PBL survival, with OS rates of 9–15 months for HIV-associated PBL [118]. According to the NCCN guidelines, the first-line treatment for HIV-associated PBL is the DA-EPOCH regimen, and CODOX-M/IVAC or R-HyperCVAD regimens are also available [74]. However, in two retrospective studies, it was found that although HIV-associated PBL had an overall response rate of up to 77% to CHOP or stronger chemotherapy regimens, survival did not improve [119-121]. Also, considering that PBL in HIV patients is more prevalent in the elderly population with poorer immune status and organ reserve, low-intensity chemotherapy regimens are preferred in the clinic [122].

Bortezomib, a proteasome inhibitor, is widely used in the treatment of multiple myeloma (MM) and mantle cell lymphoma (MCL). Based on the translocation of the immunoglobulin heavy chain gene locus in patients with HIV-associated PBL (t(8;14)), similar to those seen in multiple myeloma (t(4;14), t(14;16)) and in set cell lymphoma (t(11;14)), bortezomib also has the potential to be a therapeutic agent for PBL [120]. In HIV-associated PEL, bortezomib in combination with bortezomib alone or in combination with EPOCH has shown promising efficacy, with a 100% OR rate, a 50% 2-year survival rate, and fewer side effects [123, 124]. The CD38 monoclonal antibody daratumumab, a fully human monoclonal immunoglobulin G1, specifically targets the CD38 molecule expressed on the surface of myeloma cells and exerts antimyeloma activity through immune-mediated mechanisms, including direct induction of apoptosis and immunomodulation [125]. Based on the surface expression of CD38 in PBL, daratumumab has been investigated as a therapeutic option. In one report, a patient with HIV-associated PBL achieved CR after receiving six cycles of daratumumab in combination with EPOCH, with sustained remission for 17 months, suggesting potential efficacy of this approach [118]. Despite preliminary evidence supporting the use of bortezomib and daratumumab in HIV-associated PBL, available data remain limited to small case series and early-phase trials. Large-scale clinical trials are needed to validate efficacy, safety, and optimal dosing in this population.

### 5.5 Hodgkin Lymphoma

HIV-infected individuals are 5-15 times more likely to develop HL than the general population [126], and ART use paradoxically continues to increase this risk to 20-30 times the incidence in the general population [127]. The 5-year OS rate for HL patients with concomitant HIV infection is 14% lower than for non-HIV-infected HL patients. Although HL among HIV-infected patients is not classified as HAL in the latest version of the NCCN guidelines, understanding the treatment and management strategies for HIV-positive HL has become critical given its significantly increased prevalence in the HIV-infected population [74].

Since most HIV-positive HL patients have Ann Arbor staging of stage IV [128], a 6-cycle ABVD regimen is often used. Chemotherapy escalation is required if the early efficacy assessment Deauville score is 4-5, and the BEACOPP regimen is chosen. The 2-year PFS rate of the ABVD regimen is 80%-89%, and the 2-year OS rate is 85.7%-94%, which is not significantly different from that of HIV-negative HL patients [129, 130]. However, it is important to note that there may be drug-drug interactions between vincristine in the ABVD regimen and ART agents, such as tenofovir and atazanavir, which may cause severe hypokalaemia, proximal renal tubular dysfunction, or neurotoxicity [131-133]. The BEACOPP regimen is more suitable for patients with advanced HIV-positive HL [134]. Haemoglobin <105 g/L is one of the poor prognostic factors in advanced HL. The incidence of anaemia in HIV-positive HL was found to be about 84.8%, which far exceeds the incidence of anaemia in common HL of 40-57.4% [135, 136]. Moreover, regardless of HIV control, the rate of blood transfusion in HIV-positive HL patients is significantly higher than in HIV-negative patients (40.4% *vs*. 14.1%) [137]. This increased need may be attributed to immune dysregulation, bone marrow suppression, and other HIV-related comorbidities. Therefore, when developing treatment strategies, it is essential to address and actively manage anaemia alongside lymphoma-specific therapy in HIV-positive HL patients.

Only a small number of HIV-positive patients have been studied, so larger cohorts are needed to validate these findings. In cases where HL progresses to relapsed or refractory HIV-positive disease, treatment options include second-line chemotherapy, autologous stem cell transplantation, monoclonal anti-CD30 antibodies, and immune checkpoint inhibitors. To date, four case reports have described the efficacy of PD-1 inhibitor therapy in HIV-positive HL. Among them, two patients achieved CR and two achieved PR, including one case of relapsed/refractory disease [138-141]. Although PD-1 inhibitors appear to be safe and effective in relapsed/refractory HIV-positive HL, prospective clinical trials are required to confirm these observations.

### 5.6 Novel Materials Against HIV

The integration of heterocyclic chemistry, nanotechnology, and natural product derivatization holds immense potential for developing next-generation HIV therapies. Heterocyclic scaffolds, such as imidazo [1,2-a] pyridines and benzomorpholine derivatives, have demonstrated potent dual inhibition of HIV-1 protease (PR) and reverse transcriptase (RT) [142, 143]. For instance, morpholine-core inhibitors exhibit EC_50_ values of 2–10 nM against both HIV-1 subtypes B and C, with SAR studies revealing that electron-withdrawing substituents on the phenyl ring enhance protease binding [143]. Critical SAR insights include the importance of planar heteroaromatic rings for RT inhibition and long alkyl chains in cinnamic amides for improved membrane permeability and protease affinity [142]. While small-molecule inhibitors achieve lower EC50 values compared to nanoparticles, they often face bioavailability challenges [144].

To address these challenges, nanoparticle-based delivery systems offer a revolutionary approach to antiretroviral delivery. Lipid and protein-based nanoparticles, such as lactoferrin-curcumin co-encapsulated nanoparticles [145], enhance vaginal mucosal penetration, reducing HIV infectivity by 90% in vitro. Silica-coated calcium phosphate nanoparticles efficiently deliver DNA-based vaccines, eliciting robust humoral and cellular immune responses [146]. In comparison, lipid nanocarriers targeting activated macrophages demonstrate superior HIV reservoir penetration, achieving a 2-log reduction in viral load in preclinical models [147]. Complementing these approaches, natural agents like humic substances and protoberberine derivatives exhibit broad antiviral activity [148, 149], with humic acids inhibiting HIV-1 entry at IC50 = 5 μg/mL) and protoberberines targeting RT with IC50 values <1 μM [148, 149]. Synthetic inhibitors generally show higher specificity, whereas natural agents offer broader mechanisms but lower potency [143, 148].

However, overcoming biological barriers and clinical trial design limitations remains critical to translating these materials into lifesaving interventions. Drug resistance, particularly mutations like K103N/Y181C in RT, can diminish the efficacy of non-nucleoside inhibitors [150]. Delivery efficiency remains a challenge, with less than 20% of nanoparticle-loaded drugs reaching viral reservoirs [150]. Furthermore, only 10% of HAL patients are enrolled in immunotherapy trials due to concerns about immunosuppression [151]. Theoretical frameworks suggest that metal-organic frameworks (MOFs) could enhance drug stability via coordination bonds, a promising avenue for future research.

### 5.7 Other Treatment Options

Allogeneic hematopoietic stem cell transplantation (Allo-HSCT) has received attention as a treatment option for HAL. This is especially true for HSCT carrying the CCR5Δ32 mutation, as this mutation renders the organism resistant to HIV infection. Stem cell transplantation carrying the CCR5Δ32 gene deletion has successfully cured five cases of HIV-associated hematological cancers [152, 153]. In the 6th case of HIV successfully cured by stem cell transplantation, the donor's hematopoietic stem cells did not have a deletion of the CCR5 gene, suggesting that a CCR5Δ32 gene deletion is not necessary for achieving long-term HIV remission or cure in stem cell transplantation [154]. The 1-year OS rate for Allo-HSCT in HAL was 56.6%, and the use of Reduced Intensity Conditioning (RIC) as a pretreatment regimen was associated with a higher survival rate [155]. After transplantation, acute graft-versus-host disease (aGVHD) was reported in 59.3% of cases, with 37.5% being grade II, 6.2% being grade III, and no cases of grade IV GVHD were reported [155]. During long-term follow-up, 24 patients survived in CR and 25 died, of which 60% of deaths were related to relapse, 12% to infection, 8% to GVHD, and 20% from other causes, suggesting that Allo-HSCT has shown some efficacy in the treatment of HAL. However, some challenges remain, particularly the risks associated with GVHD and other complications.

Chimeric antigen receptor T cell (CAR-T) therapy involves genetically engineering autologous T cells to express synthetic receptors that recognize specific tumor-associated antigens. Upon reinfusion, these modified T cells bind to antigen-positive cells, activating cytotoxic signaling pathways that eliminate malignant cells. This strategy has shown efficacy in B-cell malignancies, though challenges such as cytokine release syndrome and antigen escape remain [156]. For HAL, however, available evidence is limited to case reports, since HIV-infected patients have not been included in pivotal trials. To date, six case reports have described CAR-T therapy in HAL, with three patients achieving CR and one achieving PR [156]. Although four patients developed cytokine release syndrome (CRS) or grade 3-4 immune effector cell-associated neurotoxicity syndrome (ICAN), the degree of symptoms was relatively mild. This result suggests that CAR-T cell therapy may be efficacious in patients with HAL, although immune-related adverse effects should still be monitored during treatment. As the study progresses, more data will help evaluate the long-term efficacy and safety of CAR-T therapy in the treatment of HIV-infected patients with associated lymphoma.

Traditional Chinese Medicine (TCM) enhances HAL management through immune modulation, reduction of toxicity, and direct antiviral/antitumor effects. Ganoderma lucidum improves immune reconstitution in HAART-treated patients, elevating CD4+ counts (OR: 3.21) [157]. TCM also mitigates treatment-related toxicities, as exemplified by the hepatoprotective effects of Biejiajian Pill (82.1% vs. 64.3% ALT normalization) [157]. Critically, TCM-derived agents exhibit dual activity against both HIV and lymphoma. Wogonoside suppresses tumor metastasis through ER stress–Hippo signaling [158], while Sennoside A inhibits HIV-1 replication (EC50: 0.8 μM) and integrase activity [159]. In addition, South African herbal blends have shown combined anti-HIV-1 reverse transcriptase activity (IC50: 12.5 μg/mL) and pro-apoptotic effects [160]. Real-world data from 1,752 patients demonstrated that TCM reduced viral rebound by 34% and improved ART adherence [161]. These findings highlight the potential of TCM as an adjunctive strategy, underscoring the need for standardized trials to validate its efficacy specifically in HAL.

A summary of ongoing and completed clinical trials evaluating these and other therapeutic approaches for HAL is provided in Table **3**.

## CONCLUSION

Cancer-related deaths have surpassed infection-related deaths, becoming one of the leading causes of mortality in PLWH. As the most common HIV-associated tumor, HAL has gained increasing attention [151, 162]. HAL encompasses DLBCL, BL, PEL, and PBL, which are significantly more prevalent in HIV-infected individuals and exhibit distinct clinical features and treatment strategies compared to non-HIV-related lymphomas. Treatment options for HAL include chemotherapy, monoclonal antibodies, and stem cell transplantation, though recent advances in immunotherapy and targeted therapies are reshaping clinical paradigms. DLBCL, the most common type of HAL, has traditionally utilized first-line regimens such as R-CHOP and R-EPOCH. While R-CHOP remains a cornerstone, the R+EPOCH regimen shows improved efficacy in high-risk subtypes. Notably, novel approaches like CAR-T cell therapy and bispecific antibodies have demonstrated remarkable responses in relapsed/refractory HIV-associated DLBCL, potentially influencing future first-line strategies.

For HIV-associated BL, the CODOX-M/IVAC regimen is standard, but studies exploring dose-adapted DA-EPOCH with rituximab and checkpoint inhibitors suggest reduced toxicity and enhanced efficacy. In HIV-associated PEL, despite CD20 negativity limiting rituximab utility, R+EPOCH remains first-line; however, emerging therapies targeting viral oncogenes and immune modulators are under investigation. PBL treatment prioritizes DA-EPOCH, but CODOX-M/IVAC or Hyper-CVAD are alternative options. Promi-singly, anti-PD-1 antibodies and CD30-directed therapies are being evaluated in clinical trials, reflecting a shift toward molecularly targeted interventions.

Currently, first-line treatment for HAL has reached a consensus based on antiretroviral therapy combined with chemotherapy. However, the therapeutic approach for relapsed/refractory HAL remains contentious: HSCT is constrained by the overlapping immunosuppressive risks inherent to this population, while emerging immunotherapies, such as PD-1 inhibitors and CAR-T cell therapy, lack robust validation due to the frequent exclusion of HAL patients in pivotal clinical trials. These patients face life-threatening infections driven by an immunosuppressive burden, necessitating comprehensive strategies such as intensified antimicrobial prophylaxis, IL-7-mediated immune reconstitution, and PET-CT-guided treatment de-escalation. Notably, early-phase studies of CD19-CAR-T in HAL have demonstrated encouraging complete remission rates, underscoring the need to expand clinical trial eligibility for this population. Future directions should integrate precision medicine technologies with innovative therapies targeting HIV reservoirs to achieve breakthroughs in overcoming current therapeutic barriers.

## Figures and Tables

**Table 1:** Prognostic indicators and clinical applications of HAL.

**Prognostic Indicators**	**Normal Range**	**Clinical Applications**	**References**
CD4^+^ T count	Cutoff scores: 300 cells/μl	A low CD4+ T cell count (<50 cells/μL) indicates a poor prognosis in ARL patients.	[67]
CD4+T/CD8^+^T	Cutoff scores: 0.41	A low CD4+/CD8^+^ T cell ratio is closely associated with a poor prognosis in ARL.	[68, 69]
HIV viral load	<50 copies/ml	Low viral load indicates a higher CR rate in ARL.	[66, 70]
Lymphocyte to monocyte ratio (LMR)	Cutoff scores: 3	Low LMR has a poor prognosis, with a 2-year survival rate of approximately 40.9%.	[71]
β2 microglobulin (β2-M)	serum β2-M<0.8-2 mg/L urine β2-M<0.3 mg/L	Patients with high serum β2-M levels have a 2-year survival rate of only 21.7%; high β2-M levels in urine indicate disease progression.	[72]
Hemoglobin-to-red blood cell distribution width ratio (Hb/RDW)	Cutoff scores: 8.5	Low Hb/RDW is associated with advanced Ann Arbor stage, increased extranodal sites, lower CD4 counts, higher LDH levels, and higher IPI scores.	[68]
Lactate dehydrogenase (LDH)	10-237.5 U/L	HAL with serum LDH levels > 237.5 U/L have significantly lower 3-year and 5-year survival rates; serum LDH levels exceedingly twice the upper normal limit indicate an increased mortality rate.	[73]

**Table 2:** Treatment modalities for HIV-associated lymphoma.

**Therapeutic Modality**	**DLBCL**	**BL**	**PEL**	**PBL**	**HL**
Chemotherapy	• R+EPOCH (NCCN-preferred) • R-CHOP (CD4+ >50/μL) • DA-EPOCH (3-year OS 71%)	• R-CODOX-M (2-year PFS 81%) • R-HyperCVAD (high toxicity risk)	• R+EPOCH (CR rate 58%) • MTX/ACVBP (CR 14.3%)	• DA-EPOCH (standard) • CODOX (OR 77%)	• ABVD (2-year PFS 89%) • BEACOPP (advanced HL)
Monoclonal Antibodies	• Rituximab year PFS 87.8% with CHOP; avoid if CD4^+^ <50/μL)	• Rituximab (Requires intrathecal prophylaxis)	• Rituximab (targets KSHV reservoir) • Brentuximab vedotin (CD30+ cases)	• Daratumumab (CR with EPOCH)	• Brentuximab vedotin (Salvage therapy with 75% ORR)
Hematopoietic stem cell transplantation	• Auto-HSCT (relapsed/refractoryh3-year PFS 63% in relapsed/refractoryh patients whose CD4+ >100 cells/μL)	• Auto-HSCT (limited data) - Restricted to clinical trials	• Allo-HSCT (NCT02911142): - Non-myeloablative regimens - Caution with KSHV coinfection	• Not recommended	• Auto-HSCT (relapsed/refractory): Requires sustained ART control
Immunotherapy	• Pembrolizumab (PR rate 50%) • Anti-CD19 CAR-T (CR 60%)	• CD3/CD19 bispecifics (Phase I NCT04077723)	• Nivolumab (CR in cases) • KSHV-specific T-cell therapy (preclinical)	• BCMA CAR-T (preclinical): Targets plasma cell differentiation markers	• Pembrolizumab (CR in cases) • CD30 CAR-T (Phase I NCT04288726)
Targeted therapies	• Vorinostat (targets EBV latency) • BCL-2 inhibitor Venetoclax	• Tazemetostat (MYC-driven BL) • BTK inhibitors (HIV-NHL trials)	•Lenalidomide or Pomalidomide (Downregulates IRF4 pathway) • mTOR inhibitors)	• Bortezomib (ORR 100% in small cohorts) • Selinexor (XPO1 inhibitor)	• CD30-MMAE conjugates • JAK/STAT pathway inhibitors

**Table 3:** The clinical trials of HAL.

**Conditions**	**NCT Number**	**Interventions**	**Status**	**URL**
HAL	NCT01961063	Busulfan Lentivirus vector rHIV7-shI-TAR-CCR5RZ-transduced hematopoietic progenitor cells	Active	https://clinicaltrials.gov/study/NCT01961063
ARL	NCT02797470	ASCT Carmustine	Active	https://clinicaltrials.gov/study/NCT02797470
HAL	NCT05077527	CAR-T Cyclophosphamide Fludarabine	Recruiting	https://clinicaltrials.gov/study/NCT05077527
HIV associated-DLBCL	NCT03220022	EPOCH R- EPOCH R-da- EPOCH	Recruiting	https://clinicaltrials.gov/study/NCT03220022
HAL	NCT02337985	R-EPOCH rHIV7-shI-TAR-CCR5RZ	Active	https://clinicaltrials.gov/study/NCT02337985
Untreated stage III-IV HAL	NCT01775475	CHOP ART	Finshed	https://clinicaltrials.gov/study/NCT01775475
HIV associated-DLBCL	NCT02660710	R-CHOP	Finshed	https://clinicaltrials.gov/search?cond=NCT02660710
HIV associated- BL	NCT01516593	R-CHOP HAART	Finshed	https://clinicaltrials.gov/study/NCT01516593
HAL	NCT00641381	HAART PBSC	Active	https://clinicaltrials.gov/study/NCT00641381
HIV associated- BL	NCT00392834	R-CODOX-M IVAC	Finshed	https://clinicaltrials.gov/study/NCT00392834
HAL	NCT01141712	HCT BCNU Etoposide Cytarabine Melphalan	Completed	https://clinicaltrials.gov/study/NCT01141712

## LIST OF ABBREVIATIONS

AIDS: Acquired Immunodeficiency Syndrome
ART: Antiretroviral Therapy
BL: Burkitt Lymphoma
BST2: Bone Marrow Stromal Antigen 2
CAR-T: Chimeric Antigen Receptor T Cell
CODOX-M/IVAC: Cyclophosphamide, Oncovin, Doxorubicin, Methotrexate / Ifosfamide, Vincristine, Ara-C, Carboplatin
CR: Complete Remission
CNS: Central Nervous System
DA-EPOCH: Dose-Adjusted Etoposide, Prednisone, Oncovin, Cyclophosphamide, Doxorubicin
DLBCL: Diffuse Large B-Cell Lymphoma
EBV: Epstein-Barr Virus
EPOCH: Etoposide, Prednisone, Oncovin, Cyclophosphamide, Doxorubicin
HAL: HIV-Associated Lymphoma
HHV-8: Human Herpesvirus 8
HL: Hodgkin Lymphoma
IDO: Indoleamine 2,3-Dioxygenase
IPI: International Prognostic Index
LMP1: Latent Membrane Protein 1
MOFs: Metal-Organic Frameworks
NADCs: Non-AIDS-Defining Cancers
NHL: Non-Hodgkin Lymphoma
PD-1: Programmed Cell Death Protein 1
PEL: Primary Effusion Lymphoma
PLWH: People Living with HIV
PBL: Plasmablastic Lymphoma
R-CHOP: Rituximab, Cyclophosphamide, Doxorubicin, Vincristine, Prednisone
vIRF: Viral Interferon Regulatory Factor
